# Factors affecting eye drop instillation in glaucoma patients with visual field defect

**DOI:** 10.1371/journal.pone.0185874

**Published:** 2017-10-12

**Authors:** Tomoko Naito, Koji Namiguchi, Keiji Yoshikawa, Kazuhisa Miyamoto, Shiro Mizoue, Yoichi Kawashima, Atushi Shiraishi, Fumio Shiraga

**Affiliations:** 1 Department of Ophthalmology, Okayama University Graduate School of Medicine, Okayama, Japan; 2 Department of Ophthalmology, Ehime University Graduate School of Medicine, Ehime, Japan; 3 Yoshikawa Eye Clinic, Tokyo, Japan; 4 Sumitomo Besshi Hospital, Ehime, Japan; 5 Kyoto Hitomi Care Research, Kyoto, Japan; Massachusetts Eye & Ear Infirmary, Harvard Medical School, UNITED STATES

## Abstract

**Background:**

To investigate the success rate of eye drop instillation in glaucoma patients with visual field defect as well as non-glaucoma volunteers. Factors that may affect the success rate of eye drop instillation were also evaluated.

**Design:**

A prospective, observational study.

**Participants:**

Seventy-eight glaucoma patients and 85 non-glaucoma volunteers were recruited in this study.

**Methods:**

Open angle glaucoma patients with visual field defect as well as non-glaucoma volunteers were asked to video record their procedures of eye drop instillation using a 5-mL plastic bottle of artificial tear solution. Success of eye drop instillation was judged on video based on the first one drop of solution successfully applied on the cornea, by two investigators.

**Main outcome measures:**

Success rate of eye drop instillation in glaucoma patients and non-glaucoma volunteers. Factors related to success rate of eye drop instillation, such as visual field defect and clinical characteristics, were also analyzed using multivariable logistic regression.

**Results:**

No significant deference in mean age was observed between two groups (glaucoma: 64.5 ± 14.4 years, non-glaucoma: 60.9 ± 14.1 years, *P* = 0.1156). Success rate of eye drop instillation was significantly lower (*P* = 0.0215) in glaucoma patients (30/78; 38.5%) than in non-glaucoma volunteers (48/85; 56.5%). The most frequent reason of instillation failure in glaucoma patients was touching the bulbar conjunctiva, cornea, eyelid or eyelashes with the tip of the bottle (29.5%). Multivariable logistic regression analysis identified lower corrected visual acuity (VA) (≤ 1.0; odds ratio [OR] = 0.20, 95% confidence interval [CI] 0.04–0.93, *P* = 0.0411), lower mean deviation (MD) (< -12 dB; OR = 0.20, 95% CI 0.05–0.86, *P* = 0.0307) and visual field defect (VFD) in the inferior hemifield (OR = 0.11, 95% CI 0.02–0.34, *P* < 0.001) to be significantly related to instillation failure in glaucoma patients.

**Conclusions:**

Success rate of eye drop instillation was significantly lower in glaucoma patients than in non-glaucoma volunteers. Corrected VA ≤ 1.0, MD < -12 dB and/or VFD in the inferior hemifield may be related to failure of eye drop instillation.

## Introduction

The ultimate goal of glaucoma treatment is preservation of patients’ visual function and quality of vision. To date, reduction of intraocular pressure (IOP) is the scientifically proven method for protecting the optic nerve from further damage. Recent development in glaucoma eye drops effectively decrease IOP to target level. However, sufficient IOP reduction is obtained only when the eye drops are accurately delivered onto the ocular surface. Unsuccessful instillation of eye drops may lead to treatment failure and a higher risk of disease progression. Unnecessary use of additional medications could contribute to develop local or systemic side effects.

Nonetheless, the structure of eye drop bottles has been improved in recent years, so that patients can instill one drop into their eyes accurately [1,2]. Therefore, the efficacy of topical IOP-lowering medications likely depends on the accuracy of self-instillation. Patients with advanced glaucoma who are aware of visual impairment may be more motivated to continue treatment, but these patients have greater difficulties in self-administering eye drops [3]. Such difficulties may be responsible for involuntary non-compliance not perceived by the patient [4,5]. Moreover, even when glaucoma patients have poor technique, they are often unaware of the problem [6,7].

Previous studies have reported that glaucoma patients tend to have difficulties with self-instillation of eye drops [7–9], and the factors associated with poor technique include older age [4,7,10,11], poor vision [12,13] and physical disability [14]. Stone et al [9] as well as Hennessy et al [5] evaluated the accuracy of eye drop instillation by taking videography. The rates of successful instillation of one drop onto the eye without the bottle touching the eye in patients with glaucoma and ocular hypertension were 21.9% when using a 15-mL bottle containing artificial tears or a sterile solution and 30.8% when using a 2.5-mL bottle [9]. And, the rate of successful instillation in patients with visually impaired glaucoma was 39% using a 5-mL bottle containing artificial tears [5]. However, it remains unknown how the success rate in glaucoma patients compares with that of non-glaucoma volunteers of about the same age. To the best of our knowledge, there is no videography evaluation using (1) a given bottle containing artificial tears, (2) no instruction for eye drop instillation, and (3) non-glaucoma volunteers as control.

In the present study, we judged whether one eye drop was accurately delivered on the ocular surface using video recording, and investigated the success rate of eye drop instillation in glaucoma patients compared with non-glaucoma volunteers based on the data of the video records. The factors that may be associated with the success of eye drop instillation were also analyzed.

## Participants and methods

This prospective, observational, open-label, multicenter study was approved by the Institutional Review Board of Sumitomo Besshi Hospital (UMIN registration number: UMIN 000027141). The study was conducted in accordance with ethical principles based on the Helsinki Declaration. Written informed consent was obtained from all participants after a thorough explanation of the study objective and methods. Glaucoma patients who were using one or more topical ocular hypotensive medications in one or both eyes and non-glaucoma volunteers without any apparent eye disease except mild cataract who were not using eye drops on a daily basis were recruited at each institution (Sumitomo Besshi Hospital, Okayama University Hospital, Yoshikawa Eye Clinic, and Ehime University Hospital).

### Participants

Glaucoma patients or non-glaucoma volunteers who met all of the following criteria were eligible to participate in the present study conducted from August 2012 to September 2013. Inclusion criteria for glaucoma patients were as follows: (1) 20 years or older with a diagnosis of glaucoma in one or both eyes, (2) prescribed one or more topical ocular hypotensive medications for one or both eyes at one of the institutions, (3) best-corrected visual acuity (VA, decimal visual acuity scale) ≥ 0.7, and (4) spherical equivalent refraction ≥ -10 D. Inclusion criteria for non-glaucoma volunteers were as follows: (1) 20 years or older, (2) no apparent eye diseases except mild cataract, and (3) not using eye drops on a daily basis.

Subjects were excluded if they had a history of active ocular inflammation such as recurrent uveitis, scleritis, or corneal herpes, and if they had ocular injuries, intraocular surgery, or laser surgery within 3 months before participating in the present study. We did not include subjects who had obvious disability in hand/finger motion scoring 50 points or more on the Japanese Society for Surgery of the Hand Version of the Quick Disability of the Arm, Shoulder, and Hand (QuickDASH-JSSH) Questionnaire [15]. We also excluded subjects who were judged ineligible by the investigators.

### Methods

Demographic and clinical characteristics were collected from medical records and interviews. Participants were required to demonstrate how they usually instilled eye drops using a 5-mL plastic bottle of artificial tear solution (Soft Santear, Santen, Japan). We provided facilities for the participants to instill eye drops and placed the bottle with the cap loosened on a table. Patients who were applying anti-glaucoma eye drops to one eye were asked to demonstrate the technique in that eye. Patients who were applying medication to both eyes were asked to demonstrate the technique in either eye only. No instruction was provided to participants. The examiners (K.N. and T.N.) recorded the following performance of each participant at a distance of 0.5 meter from the eye: (1) taking the bottle and opening the cap by hand, (2) bringing the bottle above an eye, and (3) squeezing the bottle and instilling solution onto the ocular surface. A digital video recorder (HDR-XR520V, Sony, Japan) at the high-resolution mode of 1,920 x 1,080/60p was used for recording the whole process of instillation. The success rate of self-instillation was compared between glaucoma patients and non-glaucoma volunteers. In addition, demographic and clinical characteristic factors related to successful eye drop instillation were analyzed in glaucoma patients and non-glaucoma volunteers.

Intraocular pressure measurement was performed using the Goldmann applanation tonometer, and visual field (VF) examination was performed using the Humphrey VF Analyzer (Carl Zeiss Meditec Inc., Dublin, CA, USA) with the standard 30–2 or 10–2 test pattern, Swedish Interactive Threshold Algorithm (SITA) standard strategy. The location of VF defects of each participant with glaucoma was determined by comparing the values of total deviation (TD). The values of the monocular TD was calculated using the Humphrey VF Analyzer for the eye in which the participant instilled eye drops. VF defect (VFD) was further divided according to the location in the superior or inferior hemifield based on the values of total deviation. The values of the monocular TD of each half was calculated separately and compared. The hemifield with lower value was considered to be the location of VFD for each participant with glaucoma, and the association between success of eye drop instillation and the location of VFD was analyzed. Additionally, we also analyzed the association between the success of eye drop instillation and the presence or absence of central VFD. Visual acuity was measured using a standard Japanese decimal VA chart, and the corrected VA was calculated using the decimal visual acuity scale.

### Criteria for judging success or failure of eye drop instillation

The success or failure of eye drop instillation was judged by two examiners (S.M. and Y.K.) who were not involved in digital video recording. When the judgement of two examiners concurred that one drop was accurately instilled onto the ocular surface at the first attempt, eye drop instillation was defined to be successful.

Meanwhile, the following situations were considered to be failure: (1) two or more attempts were required for delivering one drop onto the ocular surface, (2) two or more drops were delivered in one attempt, (3) one drop was delivered at the first attempt but the drop flowed into the conjunctival sac from the lid margin, and (4) the tip of the bottle touched the bulbar conjunctiva, cornea, eyelid or eyelashes.

### Statistical analysis

Statistical analysis was performed using JMP software version 8.0 (SAS Institute Inc., Cary, NC, USA). A *t*-test or Fisher’s exact test were used to compare normally distributed variables. A multivariable logistic regression analysis was performed to assess variables (age, gender, corrected VA, spherical power, the duration of glaucoma, the number of current eye drops, mean deviation (MD), location of VF defects, and central VF defects) that may predict success of eye drop instillation in glaucoma patients and non-glaucoma volunteers. The significance level was set at *P* < 0.05.

## Results

### Demographic and clinical characteristics of each group

A total of 163 subjects comprising 78 glaucoma patients (glaucoma group) and 85 non-glaucoma volunteers (non-glaucoma group) who fulfilled all criteria were enrolled. The demographic and clinical characteristics of each group were shown in Table 1. No significant difference (*P* = 0.1156) in mean age was observed between glaucoma patients (64.5 ± 14.4 years) and non-glaucoma volunteers (60.9 ± 14.1 years). The male to female ratio was also not significantly different between the glaucoma group (43: 35) and non-glaucoma group (36: 49) (*P* = 0.1181). The mean corrected VA was significantly lower (*P* = 0.0021) in the glaucoma group (1.01 ± 0.34) than in the non-glaucoma group (1.06 ± 0.14).

**Table 1:** Demographic and clinical characteristics of glaucoma and non-glaucoma subjects.

	Glaucoma [range]	Non-glaucoma [range]	*P*-value ^†^
**Total number of subjects**	78	85	-
**Age (years)**	64.5 ± 14.4 [32 to 88]	60.9 ± 14.1 [43 to 87]	0.1156
**Gender, number of subjects (%)**			
Male	43 (55.1)	36 (42.4)	0.1181
Female	35 (44.9)	49 (57.6)	
**Corrected VA**^‡^	1.01 ± 0.34 [0.04 to 1.5]	1.06 ± 0.14 [0.9 to 1.5]	0.0021*
**Spherical equivalent (D)**^‡^	-2.6 ± 3.6 [-12.5 to 2.5]	-0.58 ± 2.1 [-7.5 to 2.5]	0.4291
**Lens status**^‡^**, number (%)**			
Clear	30 (38.5)	58 (68.2)	< 0.001*
Cataract	9 (11.5)	17 (20.0)	0.1984
Pseudophakia	39 (50.0)	10 (11.8)	< 0.001*
**History of ophthalmic surgery, number (%)**			
Cataract surgery	39 (50.0)	10 (11.8)	< 0.001*
Glaucoma surgery	23 (29.5)	-	-
**Duration of glaucoma (years)**	5.5± 4.9 [0 to 20]	-	-
**Number of current eye drops**^§^	1.5 ± 0.9 [1 to 4]	-	-
**MD**^‡^ **(dB)**	-11.2 ± 8.9 [-31.1 to -0.01]	-	-
**Location of VF defects**^‡^ **(%)**			
Superior hemifield	49 (62.8)	-	-
Inferior hemifield	29 (37.2)	-	-
**Central VF defects**^‡^ **(%)**			
Absence	24 (30.8)	-	-
Presence	54 (69.2)	-	-

Data are shown as mean ± standard deviation.

^†^*t*-test or Fisher’s exact test.

*Statistically significant values.

^‡^Mean value of the tested eyes.

^§^Number of anti-glaucoma eye drops at video recording.

VA, visual acuity; MD, mean deviation, VF, visual field.

In the glaucoma group, the mean duration of glaucoma was 5.5 ± 4.9 years, the mean number of currently used topical ocular hypotensive medications was 1.5 ± 0.9, and the mean MD was -11.2 ± 8.9 dB. Among glaucoma patients, 62.8% had VFD in the superior hemifield, 69.2% had central VF defects, and 29.5% had a history of glaucoma surgery.

### Success or failure for eye drop instillation in each group

The success and failure rates of eye drop instillation in each group are shown in Table 2. Significantly lower (*P* = 0.0215) success rate was observed in glaucoma group (38.5%) compared to non-glaucoma group (56.5%).

**Table 2:** Success and failure rates of eye drop instillation in two groups.

	Glaucoma (n = 78)	Non-glaucoma (n = 85)	*P*-value^†^
**Success, n (%)**	30 (38.5)	48 (56.5)	0.0215*
**Failure, n (%)**	48 (61.5)	37 (43.5)	
Subjects who touched the ocular surface with the bottle	23 (29.5)	13 (15.3)	0.0374*
Subjects who required 2 or more attempts before success	18 (23.1)	17 (20.0)	0.7041
Subjects who delivered 2 or more drops in one attempt	11 (14.1)	7 (8.2)	0.3178
Subjects who delivered one drop but the drop flowed into the conjunctival sac from the lid margin	15 (19.2)	3 (3.5)	0.0196*

^†^ Fisher’s exact test.

*Statistically significant values.

Twenty-three (29.5%) of 78 patients and 13 (15.3%) of 85 volunteers touched the bulbar conjunctiva, cornea, eyelid or eyelashes with the tip of the bottle (*P* = 0.0374). Two or more attempts were required to deliver one drop onto the ocular surface in 23.1% (n = 18) of patients and 20.0% (n = 17) of volunteers (*P* = 0.7041). Eleven (14.1%) patients and 7 (8.2%) volunteers delivered two or more drops in one attempt (*P* = 0.3178). Fifteen (19.2%) patients and 3 (3.5%) volunteers delivered one drop in one attempt but the drop flowed into the conjunctival sac from the lid margin (*P* = 0.0196).

Among 18 eyes of glaucoma patients (failure cases) who made two or more attempts in eye drop instillation, one drop eventually succeeded to enter the eye in 12 eyes and failed in six eyes. Among 17 eyes of non-glaucoma volunteers who made two or more attempts, one drop was successfully delivered on the ocular surface in 16 eyes and failed in 1 eye. When cases of eye drop eventually entering the eye after multiple attempts were included in the category of “final success”, the final success rates were 53.8% (42 eyes) in glaucoma patients and 75.3% (64 eyes) in non-glaucoma volunteers. This analysis also showed that glaucoma patients were significantly less able to instill eye drops successfully (p = 0.0052, Fisher`s exact test), and the success rate of glaucoma patients did not increase as much as non-glaucoma volunteers even when success after multiple attempts was included in the analysis. In addition, we compared the number of attempts in glaucoma patients and non-glaucoma volunteers who made more than one attempt in instillation. The average number of attempts was 2.7 ± 1.4 in glaucoma patients and 2.3 ± 0.7 in non-glaucoma volunteers. Although there was with no significant difference between the two groups, glaucoma patients tended to make more attempts (p = 0.3372, t-test).

### Factors predicting the success of drop instillation in non-glaucoma volunteers

The results of the multivariate logistic regression for predicting the success of drop instillation in the non-glaucoma group are shown in Table 3. No significant differences were observed between the following subgroups: (1) age < 65 and ≥ 65 years (*P* = 0.6063), (2) corrected VA > 1.0 and ≤ 1.0 (*P* = 0.0859), (3) spherical power < -3.0 and ≥ -3.0 D (*P* = 0.8949). However, female ratio (odds ratio [OR] = 3.50, 95% confidence interval [CI] 1.29–9.49; *P* = 0.0137) reached statistical significance.

**Table 3:** Results of multivariate logistic regression analysis for predicting the success of drop instillation in non-glaucoma subjects (n = 85).

	Success Number (%)	OR	95% CI	*P*-value^†^
**Age**				
< 65 years	37/57 (64.9)	1.00	-	-
≥ 65 years	11/28 (39.3)	0.74	0.24–2.36	0.6063
**Gender**				
male	14/36 (38.9)	1.00	-	-
female	34/49 (69.4)	3.50	1.29–9.49	0.0137*
**Corrected VA**				
> 1.0	46/76 (60.5)	1.00	-	-
≤ 1.0	2/9 (22.2)	0.20	0.32–1.26	0.0859
**Spherical power**				
< -3.0 D	14/24 (58.3)	1.00	-	-
≥ -3.0 D	34/61 (55.7)	0.93	0.32–2.81	0.8949

^†^*t*-test.

*Statistically significant values.

VA, visual acuity; OR, odds ratio; CI, confidence interval.

### Factors predicting the success of drop instillation in glaucoma patients

Factors that predict success of eye drop instillation in the glaucoma group are shown in Table 4. Corrected VA ≤ 1.0 (OR = 0.20, 95% CI 0.04–0.93; *P* = 0.0411), MD < -12 dB (OR = 0.20, 95% CI 0.05–0.86; *P* = 0.0307), and VFD in the inferior hemifield (OR = 0.11, 95% CI 0.02–0.34; *P* < 0.001) were significantly associated with decreased odds of succeeding at drop instillation. There were no significant differences for age (< 65 vs. ≥ 65 years; *P* = 0.0645), gender (male vs. female; *P* = 0.6716), spherical power (< -3.0 vs. ≥ -3.0 D; *P* = 0.8434), duration of glaucoma (< 3 vs. ≥ 3 years; *P* = 0.9050), number of current eye drops (≤ 2 and > 2; *P* = 0.1119), and central VFD (absence vs. presence; *P* = 0.1957).

**Table 4:** Results of multivariate logistic regression analysis for predicting success of drop instillation in glaucoma subjects (n = 78).

	Success Number (%)	OR	95% CI	*P*-value^†^
**Age**				
< 65 years	16/32 (50.0)	1.00	-	-
≥ 65 years	14/46 (30.4)	0.20	0.04–1.11	0.0645
**Gender**				
male	16/43 (37.2)	1.00	-	-
female	14/35 (40.0)	1.29	0.39–4.20	0.6716
**Corrected VA**^‡^				
> 1.0	25/56 (44.6)	1.00	-	-
≤ 1.0	5/22 (22.7)	0.20	0.04–0.93	0.0411*
**Spherical power**^‡^				
< -3.0 D	11/24 (45.8)	1.00	-	-
≥ -3.0 D	19/54 (35.2)	0.84	0.21–6.75	0.8434
**Duration of glaucoma**				
< 3 years	9/26 (34.6)	1.00	-	-
≥ 3 years	21/52 (40.4)	1.09	0.27–4.48	0.9050
**Number of current eye drops**^§^				
≤ 2	13/38 (34.2)	1.00	-	-
> 2	17/40 (42.5)	2.80	0.79–9.9	0.1119
**MD**^‡^				
≥ -12 dB	13/29 (44.8)	1.00	-	-
< -12 dB	17/49 (34.7)	0.20	0.05–0.86	0.0307*
**Location of VF defects**^‡^				
Superior hemifield	25/49 (51.0)	1.00	-	-
Inferior hemifield	5/29 (17.2)	0.11	0.02–0.34	< 0.001*
**Central VF defects**^‡^ **(%)**				
Absence	9/24 (37.5)	1.00	-	-
Presence	21/54 (38.9)	0.40	0.09–1.60	0.1957

^†^*t*-test.

*Statistically significant values.

^‡^Mean values of the tested eyes.

^§^Number of anti-glaucoma eye drops at video recording.

VA, visual acuity; MD, mean deviation; VF, visual field; OR, odds ratio; CI, confidence interval.

## Discussion

The results of this study showed that glaucoma patients had significantly lower success rate of eye drop instillation than non-glaucoma volunteers. Multivariate logistic regression analysis revealed that the factors significantly associated with success of self-instillation in glaucoma patients were lower corrected VA, lower MD, and VF defects in the inferior hemifield (*P* < 0.05 for all).

Adherence to glaucoma eye drop use is a key factor in the management of glaucoma to prevent visual field progression [16]. To exert sufficient effect of anti-glaucoma eye drops as well as to minimize systemic and local adverse events, it is sufficient that one drop is instilled on the cornea. For confirming that one drop is accurately instilled on the cornea at the first attempt, we recorded the procedure (maneuver) of eye drop instillation by video and judged the success rate of eye drop instillation. The results of the present study revealed that 61.5% of glaucoma patients failed eye drop instillation. Even when analysis was conducted upon including cases in which eye drops were successfully delivered on the ocular surface after multiple attempts of instillation, the final failure rate remained high at 46.2%. The rate of instillation failure was apparently higher compared to those (6.8 to 20%) in previous studies [8–10,17]. The success rate of eye drop instillation in non-glaucoma volunteers was lower than we expected, probably because instillation success was judged strictly on video recording in the present study. The atmosphere of being recorded by medical professionals, in addition, may influence participants’ mental state and affect the success rate. Meanwhile, inaccurate eye drop instillation may lead physicians to believe that the current therapy is not working [4], thereby resulting in unnecessary use of additional medications. The result of this study suggests that physicians should direct interest and care on the accuracy of patient’s eye drop instillation. Of various factors that are associated with instillation failure, older age [4,7,10] has been reported to be a factor causing instillation failure. In our study, older age (≥ 65 years) was not significantly associated with instillation failure in glaucoma patients. One reason could be that we excluded glaucoma patients with obvious disability in hand/finger motion using the JSSH questionnaire in advance of the present study, and another reason is that VA is not significantly related to accurate eye drops instillation [7]. However, lower MD (< -12 dB) and VF defects in the inferior hemifield were significantly associated with lower odds of succeeding at drop instillation in the glaucoma group in the present study. Most patients with glaucoma may have preserved central VA in one or both eyes until the disease becomes advanced and affects their central vision. Difficulties with near vision tasks such as reading are the frequent complaint among patients with glaucoma who have VFD, particularly in both eyes [18–21]. Glaucoma patients with severe VFD (bilateral loss or more than a half of VF loss in either eye) had significant difficulties in glare, adjusting to bright lighting, and activities demanding functional peripheral vision [21]. Our results suggest that VF deterioration with severe glaucoma damage could be an obstacle to recognize the nozzle of the bottle and may result in low success rate of eye drop instillation.

The most frequent reason of failure for eye drop instillation in glaucoma patients in the present study was touching the bulbar conjunctiva [9], cornea, eyelid or eyelashes with the tip of the bottle (29.5%). Moreover, VFD in the inferior hemifield proved to be an independent factor in this study. These results indicate that glaucoma patients may have difficulty recognizing the correct position of the nozzle in front of an eye with inferior VFD. In such circumstances, the filling-in effect, which is an active visual process that involves creating an actual neural representation of the surroundings rather than merely ignoring the absence of information from the scotoma [22], does not help compensate the gaps in visual perception. To our knowledge, there are no clinical studies that assess how the location of VFD impacts the accuracy of self-instillation in glaucoma patients. However, Sawada et al. [23] found that lower paracentral and peripheral VF in the better eye correlated with several subscales of the 25-item National Eye Institute Visual Function Questionnaire (NEI VFQ-25), such as near vision and role limitation. Thus, glaucoma patients with VF defects in the inferior hemifield may have difficulties in near activities, which may require them to look closer at the tip of the bottle, even though their VA is preserved to a certain extent.

Our study has some limitations. First, we did not evaluate the success rate of self-instillation using various types of ophthalmic bottles. There is a possibility that the success rate may change when using different type of bottles. Second, we did not assess the history of previous education regarding the instillation technique. Previous education of drop technique was most strongly associated with good instillation technique [7]. The importance of instructing correct technique of eye drop instillation was also raised by Brown et al [6,8]. Therefore, the success rate could have been improved in glaucoma patients who had received appropriate education previously.

Despite those limitations, we recommend that the eye drop instillation technique should be assessed before adding or changing medications in glaucoma patients with VA ≤ 1.0, MD < -12 dB and/or VFD in the inferior hemifield, paying special attention to avoid touching the eye and lid with the tip of the bottle, which may cause bottle contamination [24,25] and possible serious complications such as bacterial keratitis [26]. The above-mentioned patients possibly have incorrect technique that would reduce the efficacy of the current medication. Early to moderate glaucomatous VFD usually present with asymmetric distribution between the superior and inferior hemifields. Stratifying the VF into the superior and inferior hemifields may help physicians understand patient’s difficulty to instill eye drops.

In conclusion, our results showed that glaucoma patients had significantly lower success rate of eye drop instillation than non-glaucoma volunteers. There was a possibility that the eye drop technique in glaucoma patients was influenced by VA and glaucomatous VFD. If these patients continue to have problems with the technique in spite of adequate education, concrete measures such as recommending compliance aid or asking their families to help should be taken. Improvement of their instillation technique may lead to increased drug delivery, thereby allowing the eye drops to exhibit the inherent efficacy. The present findings may help physicians recognize patients who need to be assessed on their ability to instill eye drops correctly when sufficient reduction of IOP is not obtained by current topical ocular hypotensive medications in routine glaucoma management.
